# Unique Association of Multiple Seminal Vesicle Cysts with Contralateral Renal Agenesis: A Rare Variant of Zinner Syndrome

**DOI:** 10.7759/cureus.1415

**Published:** 2017-07-01

**Authors:** Sachin Khanduri, Gaurav Katyal, Hritik Sharma, Aakshit Goyal, Nikita Singh, Harsh Yadav

**Affiliations:** 1 Radiodiagnosis, Era's Lucknow Medical College and Hospital; 2 Department of Radiology, Era's Lucknow Medical College and Hospital

**Keywords:** multiple seminal vesicle cysts, contralateral renal agensis, zinner syndrome, ejaculatory duct obstruction, infertility, renal agenesis, male counterpart of mayer-rokitansky-kuster-hauser

## Abstract

Zinner syndrome is a rare developmental anomaly of the Wolffian (mesonephric) duct which is characterised by a triad of obstruction of the ejaculatory duct, the ipsilateral seminal vesicle cyst, and the ipsilateral renal agenesis. The aim of this case report is to emphasize the importance of radiological imaging in diagnosing the condition and to report its rare unusual variant.

## Introduction

Developmental anomalies of the urogenital tract are infrequently encountered pathologies. Zinner syndrome, which is considered a male counterpart of the Mayer-Rokitansky-Kuster-Hauser (MRKH) syndrome seen in females, was first described by Zinner in 1914. It is a rare congenital urological anomaly of the mesonephric duct [1]. Fewer than 100 cases have been reported in the literature so far [2]. However, only three cases have been reported so far with contralateral renal agenesis [3-5]. We hereby report a rare case of a 40-years-old male with an unusual variant of Zinner syndrome comprising of ipsilateral ejaculatory duct obstruction and ipsilateral seminal vesicle cyst but with contralateral renal agenesis.

## Case presentation

A 40-year-old male presented to the emergency department with complaints of pain in the right lower abdomen with extension into the perineum and additional complaints of painful ejaculation. His past medical history was significant for primary infertility. On examination, the patient was afebrile and his vitals were within normal limits. An abdominal examination revealed no significant abnormality. His external genitalia appeared normal and the bilateral vas deferens were normally palpable. Lab investigations (LH, FSH, testosterone, prolactin) were within normal limits. The semen analysis showed an ejaculate volume of 1mL and oligoasthenozoospermia, a total sperm count of 9,950,000 and non-motile sperm of 98% (the normal reference range of sperm count is >20 million sperms and 2-6 mL ejaculate volume). His past surgical history was not significant.

**Figure 1:**
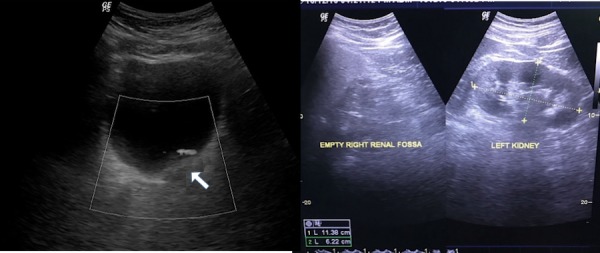
Ultrasonogram axial images In the left image, the white arrow demonstrates a bulky left-seminal vesicle whereas the right image shows an empty right renal fossa with compensatory hypertrophy of the left kidney. No ectopic kidney was found.

The patient was referred to the Department of Radio-diagnosis for a whole abdomen ultrasonography (USG) which revealed an empty right renal fossa, an enlarged left kidney, and a bulky left seminal vesicle. On the basis of the UGS, the possibility of ectopic and/or atrophic kidney was considered (Figure 1).

**Figure 2:**
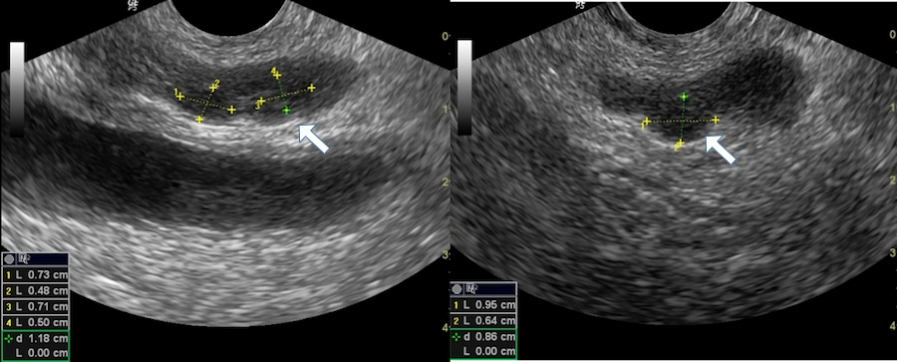
Transrectal ultrasonogram axial images The right and left images demonstrate multiple well-defined rounded hypoechoic lesions with echogenic debri within suggestive of left-sided seminal vesicle cysts with debris; the right-sided seminal vesicle is not visualised and is likely hypoplastic.

However, the exact cause of painful ejaculation was still undetermined. Following this, a transrectal ultrasound was performed under aseptic conditions which revealed multiple left-sided seminal vesicle cysts filled with echogenic debris impinging on the ipsilateral ejaculatory duct (Figure 2). The right-sided seminal vesicle was normal. The prostate and bladder were normal as well.

**Figure 3:**
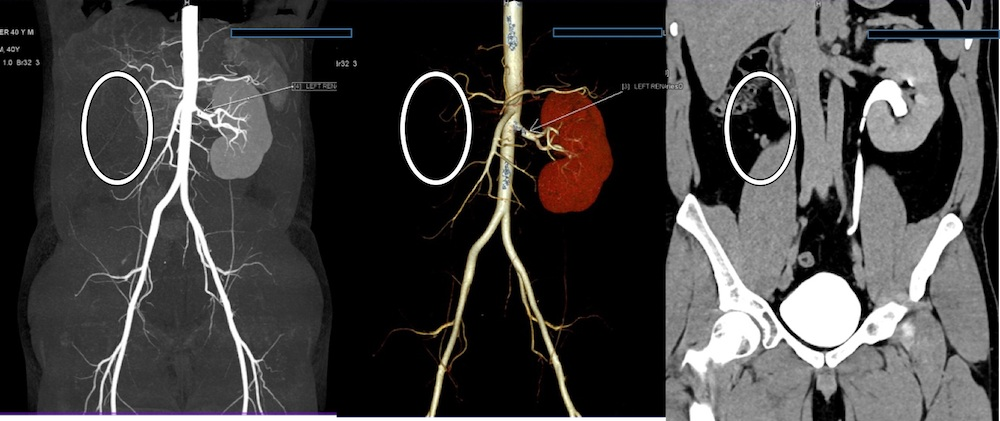
Coronal computer tomography images Maximum intensity projection (MIP), volume rendering technique (VRT), and multiplanar reconstruction (MPR) coronal images demonstrate an absent right kidney (white circle) with compensatory hypertrophy of the left kidney. The delayed MPR images show a normal course of the left ureter. No ectopic kidney was found.

**Figure 4:**
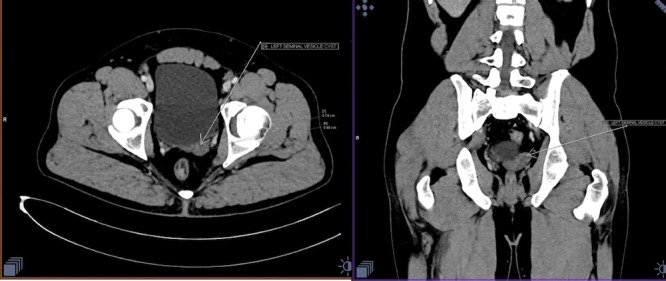
Axial and coronal multiplanar reconstruction (MPR) abdomen images The right and left images demonstrate a bulky left-seminal vesicle with multiple well-defined, non-enhancing lesions (white arrow). The right-seminal vesicle is hypoplastic.

Further investigation (contrast-enhanced computed tomography (CT) scan of the whole abdomen) was performed to confirm the above-mentioned findings. It revealed an absent right-sided kidney, absent right-sided renal artery, the vein in maximum intensity projection (MIP), and volume rendering technique (VRT). Further, delayed excretory images revealed an absent right-sided ureter. The left kidney appeared enlarged in size but with normal excretion. The left ureter appeared normal in course and calibre (Figure 3). The contrast CT revealed multiple and small well-defined hypoattenuating cysts in the region of the left seminal vesicle. The right seminal vesicle was normal (Figure 4).

## Discussion

Zinner syndrome is characterised by a distinctive triad of renal agenesis/dysplasia, ipsilateral ejaculatory duct obstruction, and ipsilateral seminal vesicle cyst. The embryological development of the genitourinary system begins at around 13th to 22nd week of gestation. The kidneys are formed as the ureteric bud interacts with the metanephric blastema. The orifice of the distal mesonephric duct and ureteric bud separates at around the sixth to the eighth gestational week [2]. The distal part of the mesonephric duct gives rise to the following structures including hemitrigone, bladder neck, urethra up to the external sphincter, s­eminal vesicle, vas deferens, ejaculatory ducts, epididymis, para-didymis, and the appendix of the epididymis under the influence of male hormone testosterone and anti-mullerian hormone. The growth factors secreted by the ureteric bud and metanephric blastema induce growth and eventually leads to the fusion of the ureteric bud with metanephric blastema. The metanephric blastema upon interaction forms the primitive kidney.

Disturbance in any of these developmental sequences can lead to the inhibition of ureteric bud growth with a failure of interaction with the metanephric blastema and subsequent fusion leading to renal agenesis. Simultaneously, the ureteric bud may fail to separate from the lower part of the mesonephric duct leading to ejaculatory duct atresia which further leads to cystic dilatation of the seminal vesicles due to poor drainage of secretions. Anomalism in the development of the distal part of the Wolffian duct causes atresia of the ejaculatory duct leading to obstruction and dilatation of the seminal vesicle while abnormal ureteral budding results in renal dysplasia/ agenesis. These developmental arrests occur in early embryogenesis [6].

It is a rare congenital disorder that accompanies other abnormalities such as polycystic renal disorders, ipsilateral testicular agenesis, atresia of the vas deferens and hemivertebrae. The frequency of ipsilateral renal malformation is higher whereas seminal vesicle cysts are seen only in 5% of the patients with renal agenesis. The incidence of seminal vesicle cyst is 0.005% and about two-third of the cases are associated with ipsilateral renal agenesis [2-3].

Although around less than 100 cases of a similar type have been documented, only four cases have been reported till date in literature with contralateral renal agenesis associated with multiple seminal vesicle cysts [4-5].

USG has been proved to be a good imaging modality in diagnosing this condition as it has high accuracy, is cheap, easily available, quick, and has no radiation risk, whereas magnetic resonance imaging (MRI) is more effective in assessing ejaculatory ducts and seminal vesicles due to its high-resolution properties [7-8].

Patients with Zinner syndrome present most commonly in the second to fourth decade of life, usually with symptoms of perineal pain, painful ejaculation, and genitourinary symptoms [9-10].

In the present case report, contrast-enhanced CT of the whole abdomen was performed which showed an absent right-sided kidney and an absent right-sided renal artery. The left ureter and left kidney were visible during the nephrogram phase and showed normal excretion of contrast in the bladder, whereas the right ureter was not visualized. Congenital seminal vesicle cyst in patients with contralateral renal agenesis is an extremely rare condition and should be considered in the diagnosis of infertility in males with renal agenesis or dysplasia.

## Conclusions

The association of renal agenesis with congenital seminal vesicle cysts is quite unusual although it is a well-documented condition. It should be considered in the differential diagnosis of cystic pelvic masses in males with renal agenesis or dysplasia. UGS is used for diagnosis; however, a CT and MRI scan can provide more accurate details. Treatment is considered only for symptomatic patients which include surgical aspiration of cysts through perineal or laparoscopic approach or percutaneous cyst drainage. Transrectal resection of the seminal vesicle cyst was advised as a definitive treatment and a close follow-up was suggested as an alternative mode. Our patient preferred the latter and is being followed up closely.
